# Supplementary material for: WASH, nutrition and health-seeking behavior during COVID-19 lockdowns: Evidence from rural Bangladesh
Source: PLoS One. 2022 Dec 7;17(12):e0278525. doi: 10.1371/journal.pone.0278525 (PMC9728864; doi:10.1371/journal.pone.0278525)
Supplement: S1 Table — (DOCX) [file pone.0278525.s001.docx]

**S1 Table. Response rate by district and socio-economic group**

|  | **Sampled** | **Interviewed** | **Response rate (%)** |
| --- | --- | --- | --- |
| **Total** | 1955 | 407 | 21% |
| **District** |  |  |  |
| Barguna | 120 | 16 | 13% |
| Jessore | 96 | 15 | 16% |
| Khulna | 440 | 96 | 22% |
| Patuakhali | 863 | 185 | 22% |
| Sathkira | 436 | 95 | 22% |
| **Socio-economic status** | |  |  |
| Poorest | 322 | 45 | 14% |
| Poor | 883 | 138 | 16% |
| Middle-income | 615 | 111 | 18% |
| Rich | 107 | 18 | 17% |
| Unregistered | 28 | 2 | 7% |
| Unknown | - | 93 | - |
