# Supplementary material for: WASH, nutrition and health-seeking behavior during COVID-19 lockdowns: Evidence from rural Bangladesh
Source: PLoS One. 2022 Dec 7;17(12):e0278525. doi: 10.1371/journal.pone.0278525 (PMC9728864; doi:10.1371/journal.pone.0278525)
Supplement: Attachment — Submitted filename: Response to Reviewers.docx [file pone.0278525.s003.docx]

Dear Reviewers,

Thank you very much for your carefully considered review of our article titled ‘WASH, nutrition and health-seeking behavior during COVID-19 lockdowns: evidence from rural Bangladesh’. We have responded to each of your comments in the table below, and made a majority of the suggested revisions. All changes can be found in the version with the ‘_Track changes’ suffix.

We hope that the updated version satisfies the reviewers’ concerns, and look forward to your feedback if additional changes are requested.

With kind regards,

Margo van Gurp

KIT Royal Tropical Institute

T +31205688564
[www.kit.nl](http://www.kit.nl)
P.O. Box 95001
1090 HA Amsterdam
The Netherlands

**Visitors address:**
Mauritskade 64
1092 AD Amsterdam
The Netherlands

**Response to reviewer 1**

**Comment 1:** Thanks to authors for presenting a study on very relevant subject: assessing impact of COVID-19 on socioeconomic activities, health, nutrition and WASH

**Response 1:** Thank you for your kind feedback and for your suggestions to improve this paper.

**Comment 2:** However, in the result section (line 201-216) it is difficult to fathom the impact of COVID-19 on breastfeeding practices. It would be good to elaborate in discussion section how the authors believe COVID-19 had any influenced breastfeeding practices

**Response 2:** Thank you for your comment, indeed the relation between breastfeeding practices and covid-19 may not be evident. We have elaborated on how breastfeeding practices could have been affected by the pandemic in lines 306 - 311 in the discussion.

**Comment 3:** Further, in the same result section how did the author capture information from parents with children of age group 2-13 months, when it was not in sampling frame as per methodology.

**Response 3:** The study was carried out approximately 10 months after the first general lockdown. The age group of 2-13 months you are referring to is the 'estimated age of the child during the lockdown' which is different from the age at time of the survey. While this is mentioned in the text, we agree with the reviewer that it could be beneficial to make this more evident. As such we indicated the age of the child at the time of the survey in text too (lines 218-221), and in the methods section (lines 147 - 148).

**Response to reviewer 2**

**Comment 1:** The study hypothesis is not clear in the introduction.

**Response 1:** Thank you for your review and valuable suggestion for improvement. We elaborated on our hypothesis in the introduction (lines 90 - 96)

**Comment 2:** It would be interesting to better describe the data collection instrument. Information is lacking to understand the variables.

**Response 2:** We have included a table highlighting the questions from the survey that were analyzed as part of this study, we hope that this provides the relevant details (Table 1).

**Comment 3:** In the data analyses, describe which was the dependent variable and which were the independent ones.

**Response 3:** The terminology 'dependent' versus 'independent' variable is specific to regression analysis, where typically one or more variables of interest (independent variables) are explored for their association with one outcome (dependent variable). However, this analysis does not describe a regression analysis but a simple test of 'independence' between two variables by means of a Chi-square test. A chi square does not distinguish between a 'dependent' and 'independent' variable. We have added an overview of key outcome variables described in this study in Table 1 of the methods section, we hope this makes it more clear.

**Comment 4:** Perform a multiple analysis to adjust for possible confounding variables.

**Response 4:** Thank you for your comment and we appreciate the effort to bring the analysis to a higher level. However, we do not believe that a multivariable analysis is in line with the scope of this study. The aim of the analysis is mainly descriptive in nature and describes many potential 'outcome variables'. We believe that a regression model (or other statistical models that allow for the adjustment of confounding and effect modification) is more appropriate when there is one or a few clearly defined outcome measures, and researchers are looking for determinants that are associated with that (set of) outcome(s). Datasets such as Demographic and Health Surveys are more suitable for this type of research.

**Comment 5:** Why were the associations made only with the variable "Socio-economic group"?

**Response 5:** We have stated the objectives of this study more clearly in the introduction (87 - 90), we hope that this addresses your question.

**Journal requirements**

1. Please ensure that your manuscript meets PLOS ONE's style requirements, including those for file naming.

**Response:** we have carefully reviewed the documents and have formatted accordingly.

2. Please include a complete copy of PLOS’ questionnaire on inclusivity in global research in your revised manuscript. Our policy for research in this area aims to improve transparency in the reporting of research performed outside of researchers’ own country or community. The policy applies to researchers who have travelled to a different country to conduct research, research with Indigenous populations or their lands, and research on cultural artefacts. The questionnaire can also be requested at the journal’s discretion for any other submissions, even if these conditions are not met.  Please find more information on the policy and a link to download a blank copy of the questionnaire here: <https://journals.plos.org/plosone/s/best-practices-in-research-reporting>. Please upload a completed version of your questionnaire as Supporting Information when you resubmit your manuscript.

**Response:** We have included said questionnaire as supporting information 2

3. PLOS requires an ORCID iD for the corresponding author in Editorial Manager on papers submitted after December 6th, 2016. Please ensure that you have an ORCID iD and that it is validated in Editorial Manager. To do this, go to ‘Update my Information’ (in the upper left-hand corner of the main menu), and click on the Fetch/Validate link next to the ORCID field. This will take you to the ORCID site and allow you to create a new iD or authenticate a pre-existing iD in Editorial Manager. Please see the following video for instructions on linking an ORCID iD to your Editorial Manager account:

**Response:** ORCID account is provided.

4. We note that Figure 1 in your submission contain [map/satellite] images which may be copyrighted. All PLOS content is published under the Creative Commons Attribution License (CC BY 4.0), which means that the manuscript, images, and Supporting Information files will be freely available online, and any third party is permitted to access, download, copy, distribute, and use these materials in any way, even commercially, with proper attribution. For these reasons, we cannot publish previously copyrighted maps or satellite images created using proprietary data, such as Google software (Google Maps, Street View, and Earth). For more information, see our copyright guidelines: <http://journals.plos.org/plosone/s/licenses-and-copyright>

**Response:** the figure is not copyrighted but created by the lead author.

5. Please review your reference list to ensure that it is complete and correct. If you have cited papers that have been retracted, please include the rationale for doing so in the manuscript text, or remove these references and replace them with relevant current references. Any changes to the reference list should be mentioned in the rebuttal letter that accompanies your revised manuscript. If you need to cite a retracted article, indicate the article’s retracted status in the References list and also include a citation and full reference for the retraction notice.

**Response:** the reference list has been reviewed.
